# Recurrent mutation in the crystallin alpha A gene associated with inherited paediatric cataract

**DOI:** 10.1186/s13104-016-1890-0

**Published:** 2016-02-11

**Authors:** Shari Javadiyan, Jamie E. Craig, Emmanuelle Souzeau, Shiwani Sharma, Karen M. Lower, John Pater, Theresa Casey, Trevor Hodson, Kathryn P. Burdon

**Affiliations:** Department of Ophthalmology, School of Medicine, Flinders Medical Centre, Flinders University, Rm 4D 111.1, Flinders Dr, Bedford Park, Adelaide, 5042 Australia; Department of Haematology and Genetic Pathology, School of Medicine, Flinders University, Adelaide, Australia; Ophthalmology Department, Women’s and Children’s Hospital, Adelaide, Australia; Mt Gambier Eye Centre, Mt Gambier, SA Australia; Menzies Institute for Medical Research, University of Tasmania, Hobart, Australia

**Keywords:** *CRYAA*, Congenital cataract, Paediatric cataract, Ion Ampliseq technologies, Next generation sequencing, PGM

## Abstract

**Background:**

Cataract is a major cause of childhood blindness worldwide. The purpose of this study was to determine the genetic cause of paediatric cataract in a South Australian family with a bilateral lamellar paediatric cataract displaying variable phenotypes.

**Case presentation:**

Fifty-one genes implicated in congenital cataract in human or mouse were sequenced in an affected individual from an Australian (Caucasian) family using a custom Ampliseq library on the Ion Torrent Personal Genome Machine. Reads were mapped against the human genome (hg19) and variants called with the Torrent Suite software. Variants were annotated to dbSNP 137 using Ion Reporter (IR 1.6.2) and were prioritised for validation if they were novel or rare and were predicted to be protein changing. We identified a previously reported oligomerization disrupting mutation, c.62G > A (p.R21Q), in the Crystallin alpha A (*CRYAA*) gene segregating in this three generation family. No other novel or rare coding mutations were detected in the known cataract genes sequenced. Microsatellite markers were used to compare the haplotypes between the family reported here and a previously published family with the same segregating mutation. Haplotype analysis indicated a potential common ancestry between the two South Australian families with this mutation. The work strengthens the genotype-phenotype correlations between this functional mutation in the crystallin alpha A (*CRYAA*) gene and paediatric cataract.

**Conclusion:**

The p.R21Q mutation is the most likely cause of paediatric cataract in this family. The recurrence of this mutation in paediatric cataract families is likely due to a familial relationship.

**Electronic supplementary material:**

The online version of this article (doi:10.1186/s13104-016-1890-0) contains supplementary material, which is available to authorized users.

## Background

Congenital or paediatric cataract is one of the major causes of lifelong visual loss in children [1] and 8–25 % of congenital cataract are inherited [2]. Inherited cataracts can be transmitted as autosomal recessive, autosomal dominant or X-linked traits, although autosomal dominant is the most common mode of inheritance. Both genetic and clinical heterogeneity have been observed and congenital cataract can occur in isolation or be associated with other systemic features as part of a metabolic or genetic syndrome [3].

Isolated inherited congenital cataracts have thus far been found to be associated with mutations in genes that encode structural proteins (including the crystallins), membrane proteins, transcription factors, signalling molecules and enzymes [4]. Crystallin genes are the largest known gene family linked with congenital cataract; mutations in several members of this gene family are known to cause the disease. More than 60 different mutations in crystallin genes have been reported to date in patients with paediatric cataracts and these mutations are associated with a range of cataract phenotypes [5, 6].

Here we aimed to determine the genetic cause of paediatric cataract in a multi-generation South Australian family using a high throughput screen of known cataract genes. We report a second occurrence of the p.R21Q mutation in the *CRYAA* gene and investigated the ancestral origins of the mutation between the two families with paediatric cataract.

## Case presentation

**Fig. 1:**
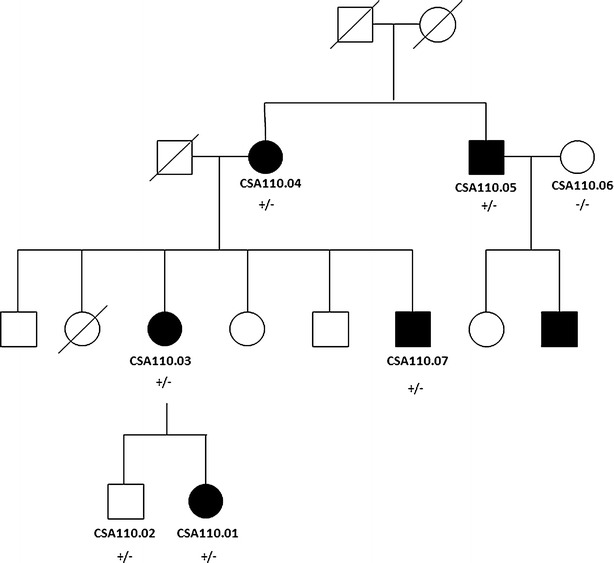
Pedigree of family CSA110 with mutation in *CRYAA.* Individuals with ID numbers were examined by an ophthalmologist. *Solid circles* indicate affected females and *solid squares* indicate affected males. *Plus sign* indicates mutant allele and *minus* *sign* indicates wild type allele. *Diagonal lines* indicate the individual is deceased

**Fig. 2:**
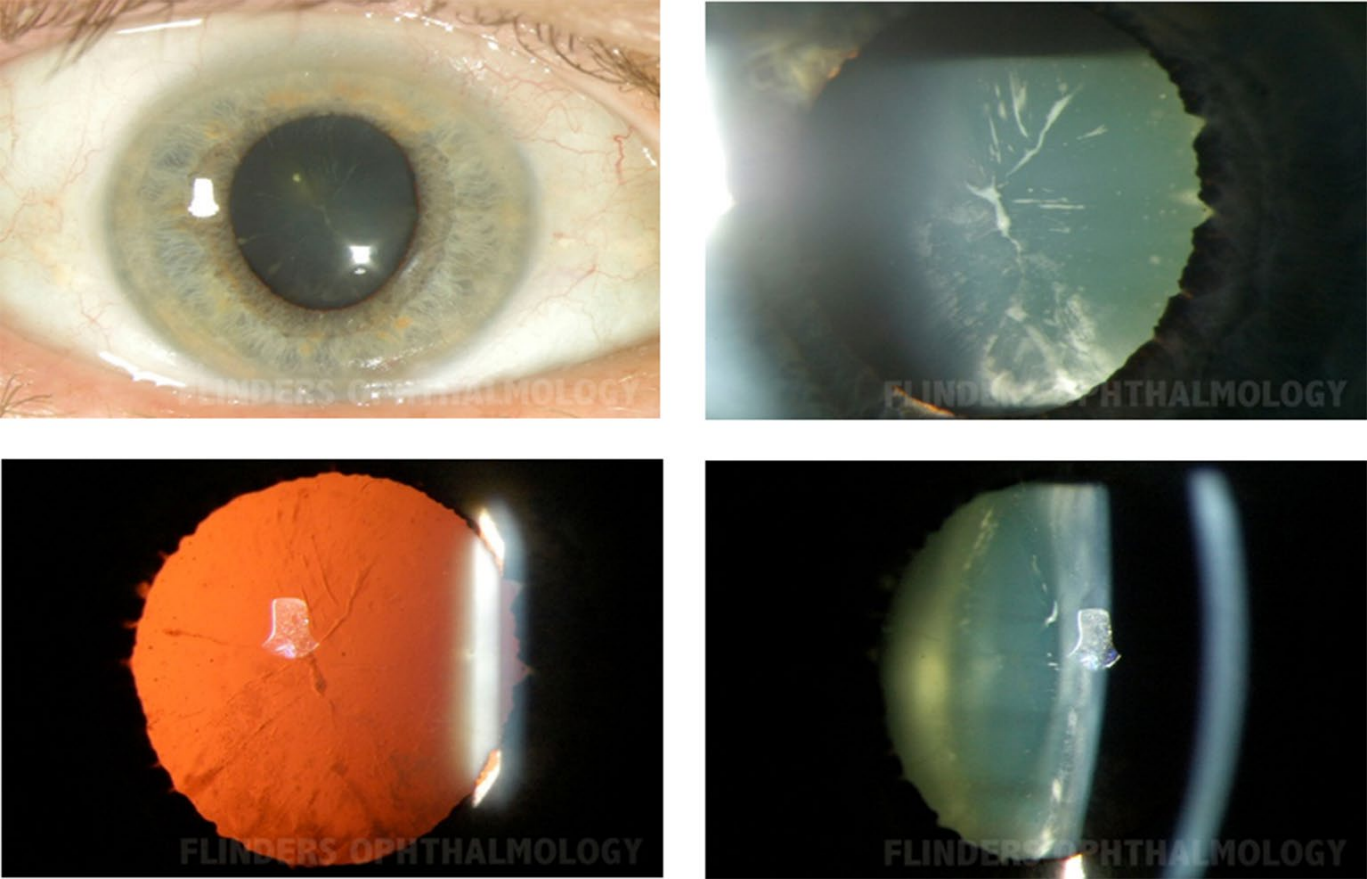
Phenotype of cataract in CSA110.05 showing lamellar and cortical cataract with *white* spokes

Six members of the three generation Australian (Caucasian) family CSA110 (Fig. 1) were diagnosed with pediatric cataract. The proband CSA110.01 presented with bilateral lamellar cataracts diagnosed at 7 years of age and had bilateral cataract surgery at age 16. Her mother, CSA110.03 also had bilateral cataracts which were diagnosed at 12 years of age and had bilateral cataract surgery at the age of 46. CSA110.07, the proband’s maternal uncle, had severe bilateral cataracts which were surgically removed at age 4 years. The proband’s grandmother, CSA110.04 had bilateral cataract surgery at the age of 76 but no information was available on the cataract phenotype. Her brother, CSA110.05, had bilateral mild lamellar cataracts, with anterior cortical spokes (Fig. 2) and had cataract surgery at age 61. These siblings had surgery significantly later than their children and grandchildren; the cataract of CSA110.05 was described as lamellar affecting the fetal nucleus indicating its congenital nature despite the late surgical intervention. The proband’s brother, CSA110.02 shows no evidence of cataracts at the last examination in 2005 when he was 20 years old.

## Discussion

Due to the heterogeneity of congenital cataract, an approach was taken that allows screening of multiple genes simultaneously. DNA (Deoxyribonucleic acid) was extracted from whole blood from all participants using the QIAamp DNA blood maxi kit (Qiagen, Hilden, Germany) according to the manufacturer’s protocol. Fifty-one genes known to cause congenital cataract in human or mouse were selected through a review of the literature and are listed in Additional file 1 [4, 7–18]. We included all the genes listed in a recent review of congenital cataract genes [4] except *gjf1* which does not appear to have a human analogue. Additional genes from more recent publications or not included in the aforementioned review were also included (Additional file 1).

PCR (polymerase chain reaction) primers to amplify coding and untranslated regions of the 51 genes were designed with the Ion Ampliseq Designer (Life Technologies, www.ampliseq.com). A total of 1216 amplicons ranging from 125 bp to 225 bp covered 94.26 % of the target sequence. Primers were supplied in two pools, 100 nM concentration each (Life Technologies, Carlsbad, CA, USA). The sequencing library was prepared with the Ion AmpliSeq library kit version 2.0 according to the manufacturer’s protocols for affected individual CSA110.03. The amplified library was diluted to 10 pM and 25 μL was used for template preparation according to Ion PGM Template OT2 200 Kit protocol using Ion PGM Template OT2 200 Kit. The clonally amplified library then was enriched on Ion OneTouch ES.

Sequencing was performed on an Ion Torrent Personal Genome Machine using The Ion PGM Sequencing 200 Kit v2 and an Ion 318 chip. Using Torrent Suite (version 3.6.) and related plugins for coverage analysis and variant calling, 1197237 reads were mapped against the reference genome (hg19), of which 93.30 % were on target. An average read depth of 918.6 was calculated for total of 1216 amplicons and 94.3 % of the target bases were covered at least 100×. Variant call format (VCF) files were uploaded to Ion Reporter V4.0 (https://ionreporter.lifetechnologies.com/ir/) for variant annotation. Variants were prioritized for further analysis if they were predicted to alter protein sequence, and were absent in dbSNP137, Exome Variant Server (EVS) (http://evs.gs.washington.edu/EVS/) and in an in-house list of sequencing errors previously seen on this gene panel. The variant is present in Exome Aggregation Consortium (ExAC) with a minor allele frequency of 0.000016.

**Fig. 3:**
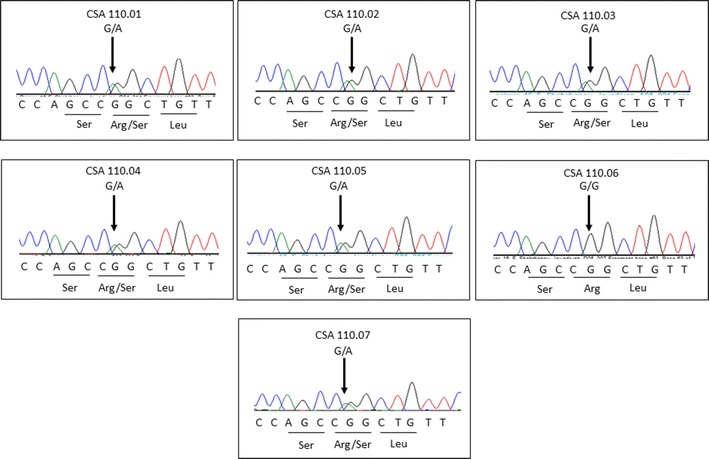
Sequence chromatogram of all examined individuals at the c.62G > A mutation. All affected family members are heterozygous, as is an unaffected individual CSA110.02

A total of 121 variants were annotated against dbSNP137, of which 1 was predicted to alter protein sequence. Interestingly, the detected mutation (c.62G > A, p.R21Q) in *CRYAA* (NM_0003941, MIM#123580) was previously shown to be a oligomerization disrupting mutation in another South Australian family independently recruited to our congenital cataract research program (Family CSA91) [19]. All the affected family members as well as one unaffected member (CSA110.02) carried the mutation as determined by Sanger sequencing (Fig. 3) using primers and methods described previously [19].

Haplotype analysis was performed in the family previously reported to carry the same missense mutation (CSA91) [19] and the new family reported here (CSA110) to determine if the mutation has arisen in both families independently, or if they are distantly related. Three microsatellite markers (D21S1260, D21S1890 and D21S1912) on the q arm of chromosome 21 flanking the *CRYAA* gene were typed in all available members of the two families. Primer sequences for amplification of each marker are provided in Additional file 2. Forward primers were labelled on the 5′ end with fluorescent dye FAM or HEX. PCR reactions of 20 μl final volume consisting of 1× Coraload PCR buffer (Qiagen) which gave a final concentration of 1.5 mM Mg^2+^, 0.1 mM dNTPs (Roche Diagnostics), 0.5 μM each primer, 0.5 U Hot Star Plus Taq Polymerase (Qiagen) and 80 ng of DNA (5× Q solution) (Qiagen) was included at a final concentration of 1× for markers D21S1890 and D21S1912. PCR was performed on a Palm cycler (Corbett Life science, QIAGEN) with 1 cycle of 95 °C for 5 min, followed by 35 cycles of 95 °C for 30 s, 57 °C for 30 s, 72 °C for 30 s and a final extension step of 72 °C for 15 min. PCR products were each diluted 1:15. Fragment analysis was carried out on a 3130xl Genetic Analyser (Life Technologies, NY USA) using GS500LIZ_3130 size standard at the Flinders Sequencing Facility (Flinders Medical Centre, Adelaide, South Australia). Fragment sizes were determined by comparing to the size standard using Peak Scanner Software Version 1 (Life Technologies). Haplotypes were reconstructed using Merlin [20]. A recombination event was detected in CSA91.05 between *CRYAA* and D21S2160.

**Fig. 4:**
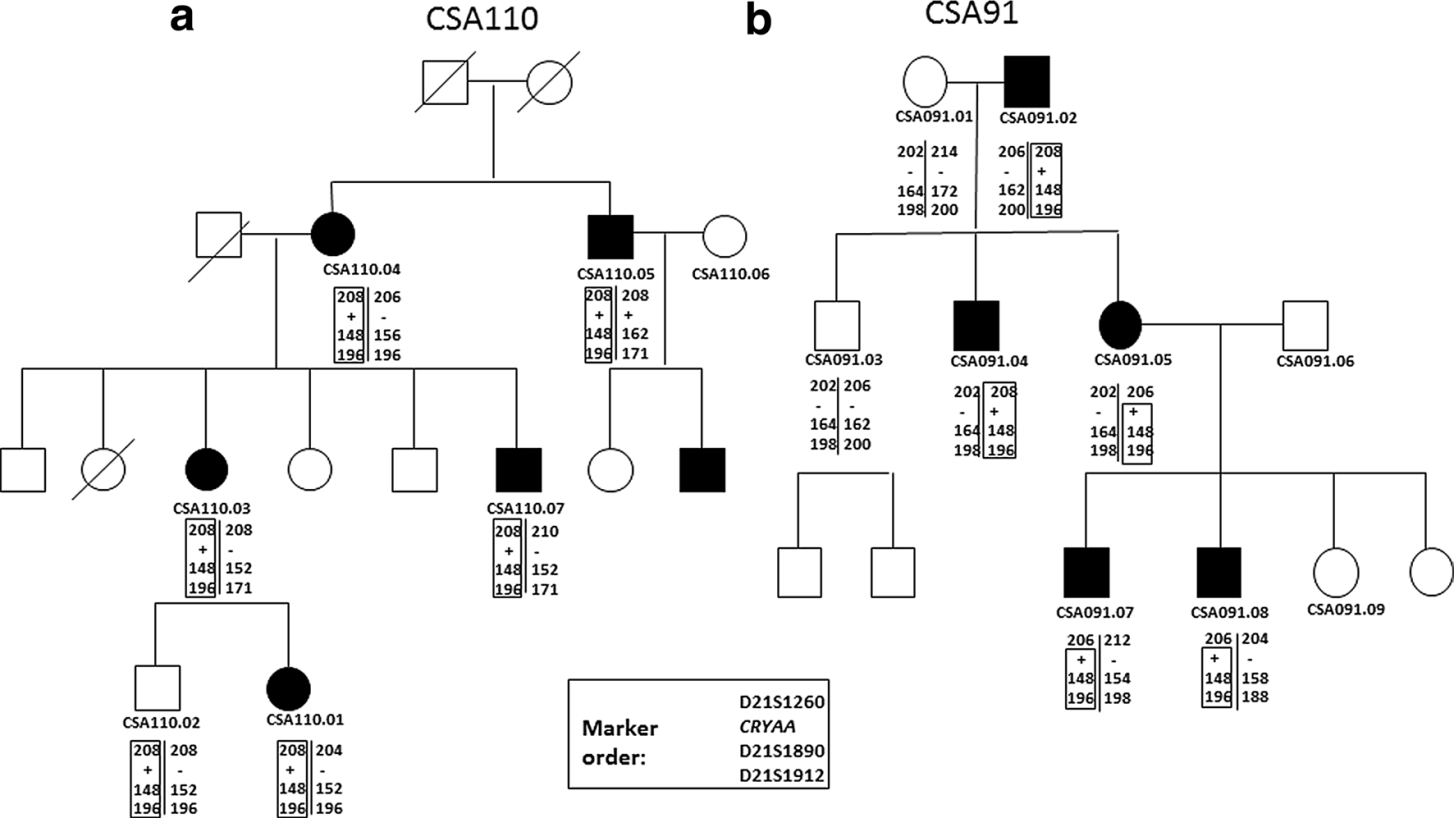
Haplotype analysis in two families with congenital cataract and the same mutation in Crystallin alpha A (*CRYAA*) a: CSA110 (reported in this study) and b: CSA91 (previously reported family). *Solid circles* indicate affected females and *solid squares* indicate affected males. Only individuals with IDs were available for study. Marker and gene order is D21S1260, *CRYAA,* D21S1890 and D21S1912 (marker names are indicated on each pedigree). Alleles at each marker are presented as the size of the PCR product detected. *Plus sign* indicates Crystallin alpha A (*CRYAA*) mutation carrier. The segregating haplotype is *boxed* and is the same in both families. A recombination event between D21S1260 and *CRYAA* was observed in CSA91.05

All of the mutation carriers in family CSA110 share a haplotype across this region of chromosome 21 with the affected family members of CSA91, indicating that the mutation has likely arisen only once on this genetic background (Fig. 4). The haplotype carrying the mutant allele was identical in the two families, suggesting that the recurrence of the mutation was possibly due to a previously unrecognised familial relationship (Fig. 4a, b).

## Conclusion

∝A-crystallin is expressed preferentially in the lens and is detectable during the early stages of lens development. It is present in high concentrations and is responsible for maintaining the transparency of the lens through chaperone like activities and by interacting with damaged or incorrectly folded proteins to prevent their precipitation [21]. It is expressed in the lens epithelium at low levels, and in the elongation zone at higher levels [22], and plays a role in the differentiation of lens epithelial cells to lens fibre cells.

Performing high throughput sequencing of 51 congenital cataract causing genes identified only one potential disease causing mutation in family CSA110. This mutation is located in *CRYAA* and segregated with the phenotype in the family. Of seven available family members, all five affected members carried the mutation, whilst one of the unaffected members also carried the mutation indicating probable reduced penetrance. Previously, the same oligomerization disrupting mutation in *CRYAA*, c.62G > A (p.R21Q), was reported in an apparently unrelated Australian family with lamellar congenital cataract of variable severity [19]. The detection of the same mutation in *CRYAA* in two families from the same state of Australia and with such a similar phenotype led us to question whether these families were related. Genetic analysis suggests that the mutation has arisen only once on the same haplotype, and therefore these families are likely to be distantly related. No genealogical link was readily identified on specific questioning. The mutation was not detected previously in Australian controls, nor was it present in commonly accessed databases at the time of sequencing, however, it has subsequently been reported in the ExAC database (http://exac.broadinstitute.org/) with a minor allele frequency of 0.000016 (i.e. 1.6/100,000 chromosomes). Thus, this variant is extremely rare, which is consistent with a role in a rare disease such as pediatric cataract.

Limitations of this study (and any amplicon based next generation sequencing platform) include missing coverage of target genes due to both limitations in oligo design and poor amplification of some amplicons. However, the *CRYAA* mutation is the most probable cause of the observed phenotype in this family as demonstrated by segregation in two families, and its reported functional effects [19]. Despite the proven advantages of exome sequencing platforms for genetic diagnosis in Mendelian and complex diseases, including congenital cataract [23–25], we decided to use a targeted sequencing approach due to its lower cost (at the time of conducting current research) and the simplified analytical pipeline for targeted re-sequencing. The findings from this study would be equally applicable to performing subset analysis of exome or genome sequencing data.

The phenotype in the previously reported family CSA91 is described as lamellar with variable severity [19] which is similar to the observed phenotype in CSA110 with variability in both severity and age at diagnosis. The inheritance pattern in CSA110 is autosomal dominant, but the mutation is not fully penetrant, in agreement with the earlier report of this mutation [13]. Since the p.R21Q *CRYAA* mutation displays both intra- and inter-familial variability, we postulate the presence of modifier genes altering the penetrance of the mutation and the severity of the cataract. There have been previous suggestions of modifier genes involved in the development of congenital cataract [26–28], although such genes have not yet been identified. Of note, mutations in *GJA3* and *CRYBB2* have been reported with reduced penetrance in congenital and pediatric cataract in humans and a digenic cause of cataract has been reported in a mouse model [28].

A phenotype of lamellar cataract with variable severity is starting to emerge in the literature for *CRYAA* mutations. To date, 11 mutations in *CRYAA* have been detected in congenital cataract patients with a variety of cataract phenotypes including lamellar cataract (Table 1). For example, Zhang et al., 2009, described a three-generation Chinese family with autosomal dominant cataract with a mutation in *CRYAA* (p.R12C). The proband was diagnosed with bilateral lamellar cataract at 1 year of age while his father has milder lamellar cataract diagnosed at 45 years of age. Other family members were diagnosed with older ages of onset of cataracts. The phenotype in this Chinese family is similar in both appearance and severity to our two Australian families.

**Table 1:** Mutations in crystallin alpha A (*CRYAA*) gene associated with pediatric or congenital cataract

Exon	DNA change	Protein change	Mode of inheritance	Reported phenotypes	References
1	c.34C > T	p.R12C	AD	Congenital, nuclear, lamellar	[25, 31–34]
1	c.61C > T	p.R21 W	AD	Congenital, laminar nuclear, polar, anterior polar	[24, 29, 31, 35]
1	c.62G > A	p.R21Q	AD	Lamellar	[19]
1	c.62G > T	p.R21L	Sporadic	Central posterior	[36]
1	c.145C > T	p.R49C	AD	Central nuclear	[29, 37]
1	c.160C > T	p.R54C	AR	Total, nuclear	[31, 38]
1	c.161G > C	p.R54P	AD	Y-sutural	[39]
1	c.161G > T	p.R54L	AD	Nuclear	[40]
2	c.292G > A	p.G98R	AD	Lamellar, punctate, Y-suture	[41, 42]
3	c.346C > T	p.R116C	AD	Zonular central nuclear, nuclear	[41, 43, 44]
3	c.347G > A	p.R116H	AD	Variable, nuclear, punctate, total	[35, 45–47]

There have been several *CRYAA* mutations reported in multiple families, although the ancestral origins have not been assessed for most. Hansen et al. [29], investigated the founder effect of a recurrent mutation (c.61C > T, p.R21 W) in 3 families using the SNP rs872331 located 55 nucleotides upstream of the mutant codon. They detected an identical haplotype c. [6T; 61C] in two of the families which confirmed their common ancestral founder with subsequent genealogical studies. The detection of the c. [6C; 61C] haplotype in the third family suggested that the mutation arose independently in that family.

**Fig. 5:**
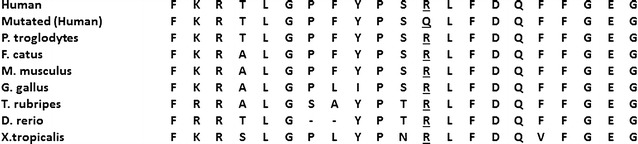
Protein sequence alignments demonstrating the conservation of the altered amino acid (*underlined*). The figure shows the alignments of crystallin alpha A protein sequence of the region of interest from seven species to the human crystallin alpha A (CRYAA) protein. Both mutated and normal human protein sequences are shown beside sequences from *Pan troglodytes* (chimpanzee), *Felis catus* (cat), *Mus musculus* (mouse), *Gallus gallus* (rooster), *Takifugu rubripes* (fish), *Danio rerio* (zebrafish) and *Xenopus tropicalis* (Frog)

The observed mutation in family CSA110 and CSA91 alters a highly conserved arginine residue at position 21 to glutamine (Fig. 5). It lies within the N terminal domain of CRYAA which has been demonstrated to be important for subunit exchange and oligomerization. It has been suggested that the deletion of this highly conserved amino acid which is located in a conserved motif (SRLFDQFFG), leads to disruption of the correct assembly of quaternary alpha-crystallin structure [30]. Western blotting of lens protein from a mutation carrier also showed a decrease in the ability of the protein to form higher order oligomers essential for its function [19]. There have been multiple reports of mutations altering the highly conserved arginine residue at position 21 (p.R21 W and p.R21L) associated with a variety of congenital cataract phenotypes, indicating its importance for proper CRYAA function.

In conclusion, we have identified a second instance of the p.R21Q mutation in the *CRYAA* gene in a family with congenital cataract and demonstrated the likelihood of these two families being distantly related. Both families showed similar phenotypes of lamellar cataracts with variable age of onset and severity, demonstrating intra- and inter-familial variability and incomplete penetrance associated with this mutation. This study highlights the local founder effects that may be seen within a community and further strengthens the genotype-phenotype correlations reported for the *CRYAA* gene with congenital cataract.

## Consent

The study adhered to the tenets of the Declaration of Helsinki and was approved by the Southern Adelaide Clinical Human Research Ethics Committee. The proband of family CSA110 (individual 110.01) was identified on presentation to the eye clinic at Flinders Medical Centre (FMC), Adelaide, South Australia. Written informed consent was obtained from all of the family members and/or their guardians for publication of this Case Report and any accompanying images. A copy of the written consent is available for review by the Editor-in-Chief of this journal. Where participants were minor (under the age of 18) or unable to personally provide consent, written informed consent was obtained from the parent or legal guardian. A detailed family history was obtained, and additional affected and unaffected family members invited to participate.

## Additional files

10.1186/s13104-016-1890-0 A table that lists reported pediatric cataract genes selected for sequencing in this study.
10.1186/s13104-016-1890-0 List of Primer sequences for the three microsatellite markers used for haplotype analysis, their position on chromosome 21 (centiMorgan) and their genomic location.

## Abbreviations

CRYAA: crystallin alpha A
PCR: polymerase chain reaction
ExAC: exome aggregation consortium
PGM: personal genome machine
