# Supplementary material for: Recurrent mutation in the crystallin alpha A gene associated with inherited paediatric cataract
Source: BMC Res Notes. 2016 Feb 11;9:83. doi: 10.1186/s13104-016-1890-0 (PMC4750205; doi:10.1186/s13104-016-1890-0)
Supplement: Supplementary file 1 — 10.1186/s13104-016-1890-0 A table that lists reported pediatric cataract genes selected for sequencing in this study. [file 13104_2016_1890_MOESM1_ESM.docx]

Additional file 1: List of reported pediatric cataract genes selected for sequencing.

| Name | Chromosome | Genbank accession | locus | Reference |
| --- | --- | --- | --- | --- |
| *GALE* | chr1 | NM_000403.3 | 1p36.11 | [[4](#_ENREF_4)] |
| *GALK1* | chr17 | NM_016905.2 | 17q25.1 | [[4](#_ENREF_4)] |
| *NSDHL* | chrX | NM_015922.2 | [Xq28](http://www.omim.org/geneMap/X/740?start=-3&limit=10&highlight=740) | [[4](#_ENREF_4)] |
| *PCBD1* | chr10 | NM_000281.3 | [10q22.1](http://www.omim.org/geneMap/10/233?start=-3&limit=10&highlight=233) | [[4](#_ENREF_4)] |
| *SORD* | chr15 | NM_003104.5 | [15q21.1](http://www.omim.org/geneMap/15/147?start=-3&limit=10&highlight=147) | [[4](#_ENREF_4)] |
| *CRYAA* | chr21 | NM_013501.2 | [21q22.3](http://www.omim.org/geneMap/21/117?start=-3&limit=10&highlight=117) | [[4](#_ENREF_4)] |
| *CRYAB* | chr11 | NM_009964.2 | [11q23.1](http://www.omim.org/geneMap/11/755?start=-3&limit=10&highlight=755) | [[4](#_ENREF_4)] |
| *CRYBA1* | chr17 | NM_009965.2 | [17q11.2](http://www.omim.org/geneMap/17/282?start=-3&limit=10&highlight=282) | [[4](#_ENREF_4)] |
| *CRYBB1* | chr22 | NM_023695.2 | [22q12.1](http://www.omim.org/geneMap/22/129?start=-3&limit=10&highlight=129) | [[4](#_ENREF_4)] |
| *CRYBB2* | chr22 | NM_007773.3 | [22q11.23](http://www.omim.org/geneMap/22/113?start=-3&limit=10&highlight=113) | [[4](#_ENREF_4)] |
| *CRYBB3* | chr22 | NM_021352.3 | [22q11.23](http://www.omim.org/geneMap/22/112?start=-3&limit=10&highlight=112) | [[4](#_ENREF_4)] |
| *CRYGA* | chr2 | NM_014617.3 | [2q34](http://www.omim.org/geneMap/2/745?start=-3&limit=10&highlight=745) | [[4](#_ENREF_4)] |
| *CRYGB* | chr2 | NM_005210.3 | [2q34](http://www.omim.org/geneMap/2/744?start=-3&limit=10&highlight=744) | [[4](#_ENREF_4)] |
| *CRYGC* | chr2 | NM_007775.2 | [2q33.3](http://www.omim.org/geneMap/2/739?start=-3&limit=10&highlight=739) | [[4](#_ENREF_4)] |
| *CRYGD* | chr2 | NM_007776.2 | [2q33.3](http://www.omim.org/geneMap/2/738?start=-3&limit=10&highlight=738) | [[4](#_ENREF_4)] |
| *CRYGS* | chr3 | NM_017541.2 | [3q27.3](http://www.omim.org/geneMap/3/740?start=-3&limit=10&highlight=740) | [[4](#_ENREF_4)] |
| *BFSP1* | chr20 | NM_001195.3 | [20p12.1](http://www.omim.org/geneMap/20/90?start=-3&limit=10&highlight=90) | [[4](#_ENREF_4)] |
| *BFSP2* | chr3 | NM_003571.2 | [3q22.1](http://www.omim.org/geneMap/3/542?start=-3&limit=10&highlight=542) | [[4](#_ENREF_4)] |
| *COL4A1* | chr13 | NM_001845.4 | [13q34](http://www.omim.org/geneMap/13/259?start=-3&limit=10&highlight=259) | [[4](#_ENREF_4)] |
| *GJA1* | chr6 | NM_000165.3 | [6q22.31](http://www.omim.org/geneMap/6/696?start=-3&limit=10&highlight=696) | [[4](#_ENREF_4)] |
| *GJA3* | chr13 | NM_021954.3 | [13q12.11](http://www.omim.org/geneMap/13/13?start=-3&limit=10&highlight=13) | [[4](#_ENREF_4)] |
| *GJA8* | chr1 | NM_005267.4 | [1q21.2](http://www.omim.org/geneMap/1/811?start=-3&limit=10&highlight=811) | [[4](#_ENREF_4)] |
| *LIM2* | chr19 | NM_005267.4 | [19q13.41](http://www.omim.org/geneMap/19/806?start=-3&limit=10&highlight=806) | [[4](#_ENREF_4)] |
| *MIP* | chr12 | NM_012064.3 | [12q13.3](http://www.omim.org/geneMap/12/435?start=-3&limit=10&highlight=435) | [[4](#_ENREF_4)] |
| *NHS* | chrX | NM_001081052.1 | [Xp22.13](http://www.omim.org/geneMap/X/94?start=-3&limit=10&highlight=94) | [[4](#_ENREF_4)] |
| *NRCAM* | chr7 | NM_001193582.1 | [7q31.1](http://www.omim.org/geneMap/7/480?start=-3&limit=10&highlight=480) | [[4](#_ENREF_4)] |
| *SPARC* | chr5 | NM_003118.3 | [5q33.1](http://www.omim.org/geneMap/5/555?start=-3&limit=10&highlight=555) | [[4](#_ENREF_4)] |
| *VIM* | chr10 | NM_203472.1 | [10p13](http://www.omim.org/geneMap/10/63?start=-3&limit=10&highlight=63) | [[4](#_ENREF_4)] |
| *FOXE3* | chr1 | NM_012186.2 | [1p33](http://www.omim.org/geneMap/1/452?start=-3&limit=10&highlight=452) | [[4](#_ENREF_4)] |
| *HSF4* | chr16 | NM_012186.2 | [16q22.1](http://www.omim.org/geneMap/16/432?start=-3&limit=10&highlight=432) | [[4](#_ENREF_4)] |
| *MAF* | chr16 | NM_005360.4. | [16q23.2](http://www.omim.org/geneMap/16/528?start=-3&limit=10&highlight=528) | [[4](#_ENREF_4)] |
| *PAX6* | chr11 | NM_000280.4 | [11p13](http://www.omim.org/geneMap/11/243?start=-3&limit=10&highlight=243) | [[4](#_ENREF_4)] |
| *PITX3* | chr10 | NM_005029.3 | [10q24.32](http://www.omim.org/geneMap/10/416?start=-3&limit=10&highlight=416) | [[4](#_ENREF_4)] |
| *SIX5* | chr19 | NM_175875.4 | [19q13.32](http://www.omim.org/geneMap/19/651?start=-3&limit=10&highlight=651) | [[4](#_ENREF_4)] |
| *SOX1* | chr13 | NM_005986.2 | [13q34](http://www.omim.org/geneMap/13/264?start=-3&limit=10&highlight=264) | [[4](#_ENREF_4)] |
| *SOX2* | chr3 | NM_003106.3 | [3q26.33](http://www.omim.org/geneMap/3/700?start=-3&limit=10&highlight=700) | [[4](#_ENREF_4)] |
| *EPHA2* | chr1 | NM_004431.3 | [1p36.13](http://www.omim.org/geneMap/1/156?start=-3&limit=10&highlight=156) | [[4](#_ENREF_4)] |
| *EFNA5* | chr5 | NM_001962.2 |  | [[4](#_ENREF_4)] |
| *AGK* | chr7 | NM_023538.2 | [7q34](http://www.omim.org/geneMap/7/605?start=-3&limit=10&highlight=605) | [[7](#_ENREF_7)] |
| *GCNT2* | chr6 | NM_008105.3 | [6p24.3-p24.2](http://www.omim.org/geneMap/6/45?start=-3&limit=10&highlight=45) | [[8](#_ENREF_8)] |
| *PVRL3* | chr3 | NM_001243288.1 | [3q13.13](http://www.omim.org/geneMap/3/421?start=-3&limit=10&highlight=421) | [[4](#_ENREF_4)] |
| *EYA1* | chr8 | NM_010164.2 | [8q13.3](http://www.omim.org/geneMap/8/278?start=-3&limit=10&highlight=278) | [[9](#_ENREF_9)] |
| *FTL* | chr19 | NM_010240.2 | [19q13.33](http://www.omim.org/geneMap/19/725?start=-3&limit=10&highlight=725) | [[10](#_ENREF_10)] |
| *CHMP4B* | chr20 | NM_176812.4 | [20q11.22](http://www.omim.org/geneMap/20/171?start=-3&limit=10&highlight=171) | [[11](#_ENREF_11)] |
| *FYCO1* | chr3 | NM_024513.3 | [3p21.31](http://www.omim.org/geneMap/3/194?start=-3&limit=10&highlight=194) | [[12](#_ENREF_12)] |
| *TMEM114* | chr16 | NM_001146336.1 | [16p13.2](http://www.omim.org/geneMap/16/142?start=-3&limit=10&highlight=142) | [[13](#_ENREF_13)] |
| *TDRD7* | chr9 | NM_014290.2 | [9q22.33](http://www.omim.org/geneMap/9/278?start=-3&limit=10&highlight=278) | [[14](#_ENREF_14)] |
| *CRYBA4* | chr22 | NM_021351.1 | [22q12.1](http://www.omim.org/geneMap/22/130?start=-3&limit=10&highlight=130) | [[15](#_ENREF_15)] |
| *VSX2* | chr14 | NM_182894.2 | [14q24.3](http://www.omim.org/geneMap/14/297?start=-3&limit=10&highlight=297) | [[16](#_ENREF_16)] |
| *PITX2* | chr4 | NM_011098.3 | [4q25](http://www.omim.org/geneMap/4/389?start=-3&limit=10&highlight=389) | [[17](#_ENREF_17)] |
| *mir 184* | chr15 | NR_038997.1 | Chr 15 | [[18](#_ENREF_18)] |
