# Supplementary material for: Recurrent mutation in the crystallin alpha A gene associated with inherited paediatric cataract
Source: BMC Res Notes. 2016 Feb 11;9:83. doi: 10.1186/s13104-016-1890-0 (PMC4750205; doi:10.1186/s13104-016-1890-0)
Supplement: Supplementary file 2 — 10.1186/s13104-016-1890-0 List of Primer sequences for the three microsatellite markers used for haplotype analysis, their position on chromosome 21 (centiMorgan) and their genomic location. [file 13104_2016_1890_MOESM2_ESM.docx]

Additional file 2: Primer sequences for the three microsatellite markers used for haplotype analysis. *CRYAA* is between D21S1260 and D21S1890 (the genomic location of *CRYAA* is chr21:44589118-44592920).

| Marker | Fluorescent label | Forward primer 5' to 3' | Reverse primer 5' to 3 | PCR product size (bp) | Position on chr21 (cM) | Genomic location (hg19) |
| --- | --- | --- | --- | --- | --- | --- |
| D21S1260 | FAM | TCCAAGGGGTTCATCC | CCCAAGGCACTGTTCC | 200-214 | 42.7 | Chr21:42796026-42796329 (21q22.3) |
| D21S1890 | FAM | GGTCTGACCACAGATTTCC | AAAAACACTCTGAACGATTAAGG | 143-173 | 44.8 | Chr21:44848156-44848388 (21q22.3) |
| D21S1912 | HEX | CCCTCATACAGATTTAAAACACAC | GAGCCCACCCTGGTAAC | 173-205 | 45.5 | Chr21:45577737-45578081 (21q22.3) |
